# Pubertal Stage, Body Mass Index, and Cardiometabolic Risk in Children and Adolescents in Bogotá, Colombia: The Cross-Sectional Fuprecol Study

**DOI:** 10.3390/nu9070644

**Published:** 2017-06-22

**Authors:** Robinson Ramírez-Vélez, Antonio García-Hermoso, Cesar Agostinis-Sobrinho, Jorge Mota, Rute Santos, Jorge Enrique Correa-Bautista, Carlos Andrés Peña-Guzmán, María Andrea Domínguez-Sánchez, Jacqueline Schmidt-RioValle, Emilio González-Jiménez

**Affiliations:** 1 Centro de Estudios para la Medición de la Actividad Física (CEMA), Escuela de Medicina y Ciencias de la Salud, Universidad del Rosario, Bogotá DC 111221, Colombia; jorge.correa@urosario.edu.co; 2 Laboratorio de Ciencias de la Actividad Física, el Deporte y la Salud, Facultad de Ciencias Médicas, Universidad de Santiago de Chile, USACH, Región Metropolitana, Santiago 7500618, Chile; antonio.garcia.h@usach.cl; 3 Research Centre in Physical Activity, Health and Leisure, Faculty of Sport, University of Porto, Porto 4200450, Portugal; cesaragostinis@hotmail.com (C.A.-S.); jmota@fade.up.pt (J.M.); rutes@uow.edu.au (R.S.); 4 Early Start Research Institute, Faculty of Social Sciences, School of Education, University of Wollongong, Wollongong, NSW 2522, Australia; 5 Facultad de Ingeniería Ambiental, Grupo de Investigación INAM-USTA Universidad Santo Tomás, Bogotá 110231426, Colombia; carpeguz@gmail.com or carlos.pena@usantotomas.edu.co; 6 Grupo de Investigación Movimiento Corporal Humano, Neuroscience Traslational Lab, Facultad de Enfermería y Rehabilitación, Universidad de La Sabana, Chia 140122, Colombia; madominguezs@unal.edu.co or mariads@unisabana.edu.co; 7 Departamento de Enfermería, Facultad de Ciencias de la Salud Avda, De la Ilustración, s/n, (18016), Universidad de Granada, Granada 18071, Spain; jschmidt@ugr.es; 8 Grupo CTS-436, Adscrito al Centro de Investigación Mente, Cerebro y Comportamiento (CIMCYC), Universidad de Granada, Granada 18071, Spain; emigoji@ugr.es

**Keywords:** pubertal stage, body mass index, obesity, cardiometabolic risk, childhood, youth

## Abstract

This study explored the association between pubertal stage and anthropometric and cardiometabolic risk factors in youth. A cross-sectional study was conducted in 2877 Colombian children and adolescents (9–17.9 years of age). Weight, height, and waist circumference were measured and body mass index (BMI) was calculated. A biochemical study was performed to determine the cardiometabolic risk index (CMRI). Blood pressure was evaluated and pubertal stage was assessed with the Tanner criteria. Hierarchical multiple regression analyses were performed. The most significant variable (*p* < 0.05) in the prognosis of cardiometabolic risk was found to be the BMI in both boys and girls. In the case of girls, the pubertal stage was also a CMRI predictive factor. In conclusion, BMI was an important indicator of cardiovascular risk in both sexes. Pubertal stage was associated with cardiovascular risk only in the girls.

## 1. Introduction

During puberty, boys and girls develop their secondary sexual characteristics and their reproductive competence, which is triggered by the secretion of the gonadotropin-releasing hormone (GnRH) [1]. Among other factors, a good nutritional status seems to be a prerequisite for a typical pubertal development [2]. Therefore, excess of adiposity can accelerate the onset of puberty and contribute to its precocious development [3]. Recent research suggests that precocious puberty is associated with immediate and long-term health risks [4,5,6].

Evidence has shown that precocious puberty is associated with a higher adult body mass index (BMI), fasting insulin, diastolic blood pressure (DBP), and decreased High-density lipoprotein (HDL) cholesterol in both sexes, as well as with higher total serum cholesterol, Low-density lipoprotein (LDL) cholesterol, and triglycerides in males [7]. Conversely, in a longitudinal study of an adolescent population, Remsberg et al. [8] found that early menarche was associated with greater cardiovascular risk as reflected in high blood pressure, glucose intolerance (regardless of age), fat-free mass, and body fat percentage.

Other studies found that excess fat (BMI and skinfold thickness) was associated with pubertal stage and with early maturation in boys and girls, though the associations were in opposite directions. Compared with their counterparts, early maturing boys had thinner skinfolds, whereas early maturing girls had thicker ones [9]. These results seem to indicate that the pubertal stage could act as a modulating factor in the development of cardiometabolic risk in young people.

The objective of this study was to explore the association between pubertal stage and biochemical and anthropometric factors in Colombian children and adolescents.

## 2. Methods

### 2.1. Study Desing and Sample Population

Schoolchildren included in this secondary analysis are part of The Fuprecol Study (Asociación de la fuerza prensil con manifestaciones de riesgo cardiovascular tempranas en niños y adolescentes colombianos, as it is called in Spanish) carried out in Bogotá, Colombia. A detailed description of Fuprecol design, methods, and primary outcomes for our current cohort can be seen elsewhere [10,11]. The present report uses a subsample (*n* = 2877) of healthy Colombian children and adolescents, 9–17.9 years of age. Data were collected from 2013 to 2016 and the analysis was conducted in 2016. The exclusion criteria included having a clinical diagnosis of cardiovascular disease, having type 1 or type 2 diabetes mellitus, being pregnant, using alcohol or drugs, and not having lived in Bogota for at least one school year. Exclusion from the study was made effective a posteriori, without the students being aware of their exclusion, to avoid any undesired situations.

### 2.2. Data Collection

Data on the variables were collected at the same time in the morning (between 7:00 a.m. and 10:00 a.m.). Body weight was measured to the nearest 0.10 kg with the participant lightly dressed using a portable electronic weight scale (Tanita^®^ Model BF689, Tokyo, Japan) with a low technical error of measurement (TEM = 0.510). Body height was measured to the nearest 0.1 cm in bare or stocking feet with the adolescent standing upright against a portable stadiometer (Seca^®^ 217, Hamburg, Germany; TEM = 0.019). Then, BMI was calculated as body weight in kilograms divided by the square of height in meters. BMI was classified as underweight, normal weight, overweight, or obese using the International Obesity Task Force (IOTF) criteria [12]. Waist circumference (WC) was measured at the midpoint between the last rib and the iliac crest using a tape measure (Ohaus^®^ 8004-MA, Parsippany, NJ, USA).

In all measures, we found almost excellent test-retest reliability (body weight, ICC, intraclass correlation, = 0.983), height (ICC = 0.973), BMI (ICC = 0.897), and WC (ICC = 0.967)). To classify WC, we used criterion-referenced health-related cut-off points derived from de Ferranti et al. [13] because of the large sample size, age-specificity, and relatively generalizable ethnicity. Body fat percentage was measured by bioelectrical impedance analysis (BIA) with a frequency current of 50 kHz using a tetrapolar BIA (Tanita^®^ Model BF689, Tokyo, Japan; TEM = 0.639) according to the protocol of Ramírez-Vélez et al. [14]. The mean of the two readings taken in the morning under controlled temperature and humidity conditions, and after urination and a 15-min rest when the participants were shoeless and fasting, was used [15].

Blood samples were obtained from each subject early in the morning, following a 10-h overnight fast by venipuncture from the antecubital vein. Before the samples were taken, the fasting condition was confirmed by the child and the parents. Blood samples were obtained from an antecubital vein, and analyses were subsequently completed within one day of collection. The cholesterol linked to high-density lipoproteins (HDL-C), glucose, triglycerides (TG), and total cholesterol were measured using colorimetric enzymatic methods with a Cardiocheck analyzer. The fraction of cholesterol linked to low-density lipoproteins (LDL-C) was calculated with the Friedewald formula [16]. The precision performance of these assays was within the manufacturer’s specifications. We calculated a cardiometabolic risk index (CMRI) as that reflects a continuous score of the five metabolic syndrome risk factors. An age-adjusted CMRI (composite z-score) was calculated for each participant using the following formula: CMRI = z-WC + z-triglycerides + z-HDL-C × (−1) + z-glucose + z-SBP + z-DBP. The HDL-C value was multiplied by −1, as it is inversely related to cardiovascular risk [17,18]. The components of the score were selected on the basis of the International Diabetes Federation [19] and modified by De Ferranti et al. [13] definitions of metabolic syndrome. All cut-off values were based on data obtained from international schoolchildren [6,19].

After the tests and blood draw, diastolic blood pressure (DBP) and systolic blood pressure (SBP) were determined as the average of two measurements separated by a five-minute interval, with the child resting for at least five minutes before the first measurement. Participants were seated in a quiet, calm environment with their right arm in a semi-flexed position at heart level. Blood pressure was measured using a Dynamap vital signs monitors (Riester Ri-Champion model, Jungingen, Germany, TEM = 0.598). High resting blood pressure for pediatric is defined as a SBP ≥ 90th centile or DBP ≥ 90th centile. All centile-based threshold limits were sex- and age-specific and selected on the basis of the De Ferranti et al. [13] definition of metabolic syndrome.

Participants self-assessed their pubertal stage of secondary sex characteristics (breast and pubic hair development for girls, genital and pubic hair development for boys; ranging from stage I to V), according to the criteria of Tanner and Whitehouse [20]. The roundup of the five composite pubertal stages were then re-classified into three pubertal stages: Tanner stage I (prepubertal); Tanner stage II–III (initiation or early puberty) and Tanner stages IV–V (mature puberty). Previous study shows moderate to high concurrence between direct physician assessment and self-assessment in this pediatric age range [21]. The reproducibility of our data reached 85%.

### 2.3. Ethics Statement

The Fuprecol Study was conducted in accordance with the Helsinki Declaration for Human Studies and approved by the Colombian Data Protection Authority (Resolution 008430/1993 Ministry of Health) and the Review Committee for Research on Human Subjects at the University of Rosario (Code No. CEI-ABN026-000262). All participants were informed of the study’s goals, and written informed consent was obtained from participants and their parents or legal guardians.

### 2.4. Statistical Analysis

The morphological component, physical fitness, and cardiometabolic risk factors of the study sample are presented as mean values, standard deviation (SD), or relative frequencies *n* (%). The normality of the variables was verified using histograms and Q-Q plots (“Q” stands for quantile). Differences were analyzed with the Student *t*-test or chi-square test (χ^2^) in order to explore differences between sex and pubertal stage groups. The ANCOVA model was used to assess mean differences in CMRI among the categories of pubertal stage, controlling for age.

Each of the component variables of the risk score was regressed with age (and with body height for SBP and DBP) separately for boys and girls. The standardized residuals were retained to represent the z-score of age-adjusted values for each of the component variables. All the statistical analyses were conducted separately for boys and girls. In parallel, a CMRI without the central obesity component (i.e., WC) was also calculated for comparison. Hierarchical multiple regression analyses were applied to examine the interaction effect of pubertal stage on the association between CMRI and BMI. The z-score of the unadjusted BMI (ZBMI) was first entered into the regression model with the CMRI score as the dependent variable. Then, pubertal stage was recoded as a categorical variable (pubertal and post-pubertal with pre-pubertal as a reference) and entered into the regression. Finally, the interaction terms of the pubertal stages and ZBMI were entered into the regression model. The *F*-test was used to assess the significance of the additional terms in comparison to the preceding model. The interaction effect of the pubertal stage was indicated by the significance of the interaction terms added to the regression model. All models were adjusted for age. We used SPSS V. 21.0 software for Windows (SPSS, Chicago, IL, USA). Statistical significance was set at *p* < 0.05.

## 3. Results

### 3.1. Descriptive Characteristics

The sample population was composed of 2877 participants, 54.5% of whom were female. No statistically significant gender-based differences were found (*p* = 0.067). As can be observed in Table 1, the nutritional status of the subjects showed statistically significant differences depending on sex (*p* = 0.001), with a higher prevalence of overweight (23.1%) in the girls. When the amount of body fat was analyzed, the results showed that the girls had an average of 24.9% whereas the boys had an average of 16.6%. These differences were statistically significant (*p* = 0.013).

Regarding stages of pubertal stage, there were no significant differences between boys and girls (*p* = 0.157).

### 3.2. Pubertal Stage and Cardiovascular Risk Factors

Table 2 shows differences between pubertal stage categories in cardiovascular risk factors. The variables showing statistically significant differences (*p* < 0.05) were high systolic blood pressure, overweight/obese and obese in both sexes. Moreover, in the case of the boys, there were statistically significant differences in the variables of low HDL-C (*p* = 0.001) and CMRI (*p* = 0.003). As can be observed, in the post-pubertal stage, CMRI values were positive in both sexes.

Summary risk scores to quantify the cardiovascular risk were used to examine the interaction effect of pubertal stages on the association of cardiovascular risk factors by BMI in the analyses. When sex-specific characteristics for the CMRI, the ZBMI explained a significant proportion of the variance (*R*^2^) in CMRI in both boys (20.0%) and girls (20.5%). Pubertal stage was entered in Model 2 and accounted for a significant increase in variance explained in both sexes, (boys *R*^2^ = 22.0% and girls *R*^2^ = 21.0%). When the interaction-term of ZBMI and puberty was entered in Model 3, there was a further significant increase of the variance explained in both sexes, (boys *R*^2^ = 23.0% and girls *R*^2^ = 23.0%) (Table 3, CMRI ^a^). On the other hand, hierarchical regression results for the CMRI without the central obesity component (i.e., WC), shown a lower proportion of variance explained in each model.

In particular, the marginal effect of increasing 1 unit of ZBMI on the CMRI would be further increased by an average of 0.016 and 0.009 units to the score for girls in the pubertal stage and post-pubertal stage, respectively (Table 3, CMR1 ^a^). Such an interaction effect of pubertal stage was, however, reversed in boys; the marginal effect of increasing 1 unit of ZBMI on the CMRI was decreased by an average of 0.050 and 0.011 units in the pubertal stage and post-pubertal stage, respectively (Table 3). A similar pattern of the interaction effect of pubertal stage on the association between ZBMI and CMRI without the central obesity component was observed (Table 3, CMRI ^b^).

## 4. Discussion

This study explored the association between pubertal stage and anthropometric and cardiometabolic risk factors in 2877 Colombian children and adolescents (9–17.9 years old). Our findings show significant gender-based differences in the nutritional status of the participants with a higher prevalence of overweight among the girls (23%). This finding agrees with Briceño et al. [22], who studied a population of children and adolescents in Bogotá and found that there were 18.2% more overweight girls than boys.

Previous studies have shown that the timing of pubertal development affects body composition in girls [22] and in boys [6]. In line with the findings of Caicedo-Álvarez et al. [11], who studied a population of Colombian children living in the city of Bogotá, we also found (as expected) that mean WC values increased with age. Moreover, studies have shown that the distribution pattern of fat varies with age [2,6,7,8,9], with a tendency for fat to be deposited in the central area of the body instead of in peripheral areas, which heightens the risk of cardiovascular disease [7]. In same line, we have found a higher rate of increased overweight/obese among pre-pubertal boys with a decreasing trend towards pubertal/post-pubertal boys. However, prevalence of overweight/obese among the girls was greater than that among boys as compared to pubertal stage. These results also coincide with those obtained by Freedman et al. [23], who conducted a prospective study of 6866 boys and girls, 5–17 years of age, in Louisiana (USA). In that study, the author found that the BMI values of girls were considerably higher than those of boys. Furthermore, the tendencies regarding obesity status reported in our study are also in consonance with the general statistics of Colombia’s 2016 Report Card on physical activity for children and youth [24]. These research results justify the need to implement programs that foment healthy life styles from an early age. Moreover, the analysis of body composition in our study found significant differences in the body fat percentages in both sexes with a higher prevalence in girls (24.9%) in comparison to boys (16.6%).

According to Prenkert and Ehnfors [25], these differences in body fat percentage could be due to differences in physical and sexual development, which generally occur earlier girls. Therefore, before puberty, from five up to the age of ten, both sexes have similar amounts of body fat. However, during puberty, girls usually experience an increase in body fat whereas in boys, there is a decrease [26]. For this reason, scientific evidence suggests that pubertal development affects body composition in both sexes [27,28,29].

In regard to pubertal stage and cardiovascular risk factors, puberty was significantly associated with some cardiovascular risk factors, such as high systolic blood pressure, overweight/obesity, and obesity. This suggests that the simple and inexpensive measure of BMI can be as clinically important or even more important than total adiposity measures assessed by accurate, complex, and expensive methods [30]. Nonetheless, the results showed that the most significant variable in the prognosis of cardiometabolic risk was BMI. In addition, the Tanner pubertal development stages seem to be a predictor of cardiometabolic risk [6].

One of the major issues at puberty is the difference in the percentage of body fat between sexes. Consequently, for girls, the physiological pattern of body fat, running parallel to age and pubertal development (especially late/post-pubertal development), could be associated with increased early cardiovascular risk [6]. As for the boys, in addition to the previously mentioned factors, puberty was significantly associated with low HDL-C and CMRI. Therefore, our findings indicate that pubertal development (i.e., late/post-pubertal) can be a determining factor in the predisposition to cardiometabolic risk, especially in girls, which coincides with the results of previous studies [31,32]. Nonetheless, the findings obtained in other studies [31] were somewhat less conclusive regarding the association of pubertal development stages and cardiometabolic risk in boys, but still agree with the results of our study.

We explored the association between pubertal stages and individual cardiometabolic risk factors to examine whether pubertal stage was associated with CMRI, whereas the other studies looked into the changes of metabolic profile that occur in different pubertal stages by sex. Additionally, the association of CMRI is statistically more demanding than CMRI, as it removes the WC component from the risk score. The results of our study as well as those of other studies suggest that it would be appropriate to include the evaluation of pubertal development stage in clinical screening, given its association with cardiovascular risk. However, similar to the findings of the Chan et al. [6] in Hong Kong Chinese children, the increases in the variance explained by the pubertal stage (i.e., interaction-term) was negligible (1%–2%), so these data should be cautiously interpreted. Therefore, further research is necessary to identify and monitor the factors that lead to higher adiposity levels in children and adolescents in Bogotá. In fact, these results should be a wake-up call for the Colombian government since they are a clear justification of the need to implement policies that foment healthy lifestyles in young people. This includes regular physical exercise, as well as a balanced diet in early childhood. Schools may be an ideal setting to monitor cardiometabolic risk factors [24,30,31,32] and to formulate and apply specific strategies to promote young people’s health.

This study had some limitations. First, all participants were from the same region in Colombia. Consequently, any inferences for all Colombian children and adolescents should be made with caution. Second, this research did not consider the potential impact of determinants such as socioeconomic, dietary and physical activity patterns, and ethnic factors, all of which can modulate growth and levels of adiposity. However, such limitations do not compromise the interest of the results obtained. One of the strong points of the study is its large sample size. Another positive aspect is that the children and adolescents in the sample attended public schools, which were located in different districts of Bogotá, therefore including a mix of locally-born residents and those arriving from other regions, and so it is racially and culturally diverse.

## 5. Conclusions

Our findings suggest that there may be an interaction effect of pubertal stage on the association of cardiometabolic risk by BMI. Nevertheless, further research is required to analyze in greater depth the association between different pubertal stages and risk factors such as obesity at early ages. These results can be used as a baseline for long-term health surveillance. A longitudinal study is needed to further examine any moderation effect.

## Figures and Tables

**Table 1:** Characteristics among a sample of children and adolescents from Bogota, Colombia (mean (SD) or frequencies).

Characteristics	Overall (*n* = 2877)	Girls (*n* = 1568)	Boys (*n* = 1309)	*p* Value
Age (years)	13.2 (2.2)	13.2 (2.2)	13.3 (2.3)	0.067
Height (cm)	152.9 (11.9)	150.7 (9.5)	155.6 (13.8)	<0.001
Body mass (kg)	46.8 (11.6)	46.2 (10.7)	47.6 (12.5)	<0.001
Body mass index (kg/m^2^)	19.8 (3.1)	20.1 (3.2)	19.3 (2.9)	0.002
Weight status *n* (%) ^a^				
Underweight	446 (15.5)	243 (15.5)	203 (15.5)	0.021
Normal	1728 (60.1)	861 (54.9)	867 (66.2)	
Overweight	522 (18.1)	362 (23.1)	160 (12.2)	
Obesity	181 (6.3)	102 (6.5)	79 (6.0)	
Waist circumference (cm)	64.6 (7.6)	63.8 (7.5)	65.7 (7.6)	0.872
Body fat BIA (%)	21.1 (7.5)	24.9 (6.0)	16.6 (6.4)	0.013
Pubertal stage *n* (%) ^a^				
Pre-puberty	163 (5.7)	78 (5.0)	85 (6.5)	0.157
Puberty	1473 (51.2)	794 (50.6)	679 (51.9)	
Post-puberty	1241 (43.1)	696 (44.4)	545 (41.6)	
SBP (mmHg)	111.7 (13.3)	110.3 (12.4)	113.3 (14.0)	<0.001
DBP (mmHg)	68.4 (8.9)	68.5 (8.6)	68.1 (9.3)	0.282
Total cholesterol (mg/dL)	145.0 (31.5)	149.4 (30.6)	139.6 (31.6)	0.504
HDL cholesterol (mg/dL)	47.2 (12.3)	47.4 (12.2)	46.8 (12.4)	0.226
LDL cholesterol (mg/dL)	83.3 (31.1)	85.1 (28.3)	81.7 (34.3)	<0.001
Triglycerides (mg/dL)	91.3 (48.4)	96.5 (54.1)	85.4 (39.4)	0.001
Glucose (mg/dL)	82.4 (15.8)	81.5 (15.7)	83.3 (15.7)	0.806

Sex differences were analyzed with the Student *t*-test (means (SD)) or ^a^ chi-square test (χ^2^) for *n* (%). BIA= Bioelectrical impedance analysis.

**Table 2:** Association between pubertal stage and cardiometabolic risk factors by sex.

Characteristics	Girls (*n* = 1568)			*p* Value	Boys (*n* = 1309)			*p* Value
	Pre-Pubertal (*n* = 78)	Pubertal (*n* = 794)	Late/Post-Pubertal (*n* = 696)		Pre-Pubertal (*n* = 85)	Pubertal (*n* = 679)	Late/Post-Pubertal (*n* = 545)	
Age (years) (mean (SD)) ^a^	10.3 (1.4)	12.4 (2.1)	14.4 (1.7)	0.001	10.2 (1.8)	12.4 (2.1)	14.6 (1.6)	0.001
Cardiometabolic risk factors (*n* (%))								
Increased waist circumference	3 (3.8)	28 (3.5)	23 (3.3)	0.765	4 (4.7)	29 (4.3)	22 (4.0)	0.660
High triglyceride	19 (24.4)	258 (32.5)	233 (33.5)	0.231	12 (14.1)	142 (20.9)	127 (23.3)	0.130
Low HDL-C	45 (57.7)	497 (62.6)	458 (65.8)	0.065	39 (45.9)	383 (56.4)	404 (74.1)	0.001
High fasting plasma glucose	1 (1.3)	30 (3.8)	40 (5.7)	0.200	2 (2.4)	39 (5.7)	28 (5.1)	0.824
High systolic blood pressure	19 (24.4)	117 (14.7)	69 (9.9)	<0.001	17 (20.0)	117 (17.2)	68 (12.5)	0.004
High diastolic blood pressure	12 (15.4)	91 (11.5)	71 (10.2)	0.200	12 (14.1)	63 (9.3)	56 (10.3)	0.556
Overweight/obese	22 (28.2)	220 (27.5)	209 (30.0)	0.010	27 (31.8)	118 (17.4)	82 (15.0)	0.006
Obese	4 (5.1)	54 (6.8)	36 (5.2)	0.006	8 (9.4)	39 (5.7)	23 (4.2)	0.005
CMRI (mean (SD)) ^b^	−0.053 (0.512)	−0.025 (0.506)	0.005 (0.464)	0.392	−0.087 (0.421)	−0.043 (0.495)	0.038 (0.480)	0.003

*p* value testing the statistical significance of the association between each of the cardiometabolic risk factors and pubertal stage. CMRI, cardiometabolic risk index. CMRI = z-WC + z-triglycerides + z-HDL-C + z-glucose + z-SBP + z-DBP. The HDL-C value was then multiplied by −1, as it is inversely related to cardiovascular risk. We used a lineal chi-square test (χ^2^) for *n* (%). ^a^ Analyzed by ANOVA one-way (*p* for trend); ^b^ Analyzed by ANCOVA model adjusted by age.

**Table 3:** Unstandardized regression coefficients examining the association of pubertal stage and cardiometabolic risk score adjusted by age.

**CMRI ^a^**	**Girls (*n* = 1568)**					**Boys (*n* = 1309)**				
	**B**	**SE**	***p***	**Adjusted *R*^2^**	***p* Value (∆F)**	**B**	**SE**	***p***	**Adjusted *R*^2^**	***p* Value (∆F)**
Model 1										
Intercept	−0.020	0.011	0.076	20.5	<0.050	−0.010	0.012	0.414	20.0	<0.050
ZBMI	0.270	0.014	<0.050			0.225	0.013	<0.05		
Model 2										
Intercept	0.302	0.066	<0.050	21.0	<0.050	0.026	0.068	0.704	22.0	<0.050
ZBMI	0.290	0.015	<0.050			0.226	0.013	<0.05		
Puberty	−0.053	0.018	0.003			−0.008	0.016	0.599		
Post-puberty	−0.084	0.016	<0.050			−0.008	0.017	0.628		
Model 3			<0.050							
Intercept	0.316	0.067	<0.050	22.0	<0.050	0.050	0.069	0.462	23.0	<0.050
ZBMI	0.331	0.080	<0.050			0.395	0.069	<0.050		
Puberty	−0.064	0.020	0.001			−0.010	0.017	0.541		
Post-puberty	−0.084	0.016	<0.050			−0.017	0.016	0.293		
ZBMI × Puberty	0.016	0.020	0.011			−0.050	0.017	0.004		
ZBMI × Post-puberty	0.009	0.021	0.665			−0.011	0.017	0.507		
**CMRI ^b^**	**Girls (*n* = 1568)**					**Boys (*n* = 1309)**				
	**B**	**SE**	***p***	**Adjusted *R*^2^**	***p* Value (∆F)**	**B**	**SE**	***p***	**Adjusted *R*^2^**	***p* Value (∆F)**
Model 1										
Intercept	−0.016	0.008	0.048	13.9	<0.050	−0.009	0.069	0.414	9.1	<0.050
ZBMI	0.115	0.012	<0.050			0.156	0.083	<0.05		
Model 2										
Intercept	0.125	0.046	<0.050	14.5	<0.050	0.019	0.028	0.309	11.3	0.051
ZBMI	0.209	0.010	<0.050			0.226	0.036	<0.050		
Puberty	−0.024	0.009	<0.001			−0.008	0.039	0.599		
Post-puberty	−0.059	0.011	<0.050			−0.008	0.049	0.628		
Model 3			<0.050							
Intercept	0.230	0.046	<0.050	15.1	<0.050	0.050	0.124	0.340	11.9	<0.050
ZBMI	0.292	0.056	<0.050			0.395	0.189	<0.050		
Puberty	−0.041	0.012	<0.001			−0.010	0.030	0.569		
Post-puberty	−0.063	0.010	<0.050			−0.017	0.096	0.203		
ZBMI × Puberty	0.008	0.012	<0.050			−0.050	0.083	<0.050		
ZBMI × Post-puberty	0.002	0.013	0.366			−0.011	0.070	0.507		

ZBMI: Body mass index (BMI) z-score; B: Unstandardized regression coefficient; SE: Standard error; *p*: *p*-value testing the significance of the regression coefficient; *R*^2^: Variance explained by the regression model-adjusted values; *p* (∆F): *p*-value testing the significance of *F* change from the preceding model (model 1 includes only the intercept term); ref: reference group of the categorical variable analyzed by creating dummy variables; ^a^ CMRI = z-WC + z-triglycerides + z-HDL-C + z-glucose + z-SBP + z-DBP. The HDL-C value was then multiplied by −1, as it is inversely related to cardiovascular risk; ^b^ CMRI includes all components of risk score (a) except waist circumference (WC).

## Abbreviations

The following abbreviations are used in this manuscript:

BIA	bioelectrical impedance analysis
BMI	body mass index
CMRI	cardiometabolic risk index
DBP	diastolic blood pressure
ENSIN	National Survey on the Nutritional Situation
GnRH	gonadotropin-releasing hormone
HDL-C	high-density lipoprotein cholesterol
IOTF	International Obesity Task Force
LDL-C	low-density lipoprotein cholesterol
SBP	systolic blood pressure
TC	total cholesterol
TG	triglycerides
WC	waist circumference
